# Development of Sustainable Cement Asphalt Mortar Using Agricultural Waste-Derived Bio-Oil and Latex–Acrylic Polymers for Enhanced Durability

**DOI:** 10.3390/polym16223210

**Published:** 2024-11-19

**Authors:** Yeong-Min Kim, Kyungnam Kim, Tri Ho Minh Le

**Affiliations:** 1 Department of Highway & Transportation Research, Korea Institute of Civil Engineering and Building Technology, 283 Goyangdae-ro, Ilsanseo-gu, Goyang-si 10223, Gyeonggi-do, Republic of Korea; choozang@kict.re.kr; 2 Korea Expressway Corporation Research Institute, Pavement Research Division, Dong-tansunhwan-daero 17-gil, Hwaseong-si 18489, Republic of Korea; 3 Faculty of Civil Engineering, Nguyen Tat Thanh University, 300A Nguyen Tat Thanh Street, District 4, Ho Chi Minh City 700000, Vietnam

**Keywords:** cement asphalt mortar, bio-oil, polymer additives, sustainability, chemical resistance, thermal stability

## Abstract

Cement Asphalt Mortar (CAM) is widely applied in infrastructure, particularly in railways, bridge expansion joints, and pavements, due to its combination of cement’s load-bearing capacity and asphalt’s flexibility. Conventional CAM formulations, however, often encounter challenges such as extended setting times, high shrinkage, and limited durability under extreme environmental conditions. This study addresses these limitations by integrating bio-oil and polymer additives to enhance both the sustainability and performance of CAM mixtures. CAM mixtures were evaluated with cement-to-asphalt emulsion (C/AE) mass ratios of 75:25 and 50:50, incorporating bio-oil contents of 2% and 4% by mass and latex–acrylic polymer proportions ranging from 1% to 2% by mass. The optimized mix design, with a 75:25 cement-to-asphalt emulsion (C/AE) mass ratio, 2% bio-oil, and 1.5% polymer, improved flowability by 25%. This formulation achieved a flow diameter of approximately 205 mm and reduced the flow time to 72 s. Compressive strength tests indicated that this formulation reached an early-stage strength of 10.45 MPa (a 20.8% improvement over the control) and a 28-day strength of 24.18 MPa. Thermal stability tests at 45 °C demonstrated that the optimized CAM retained 86.6% of its compressive strength, compared to a 25% reduction in unmodified mixtures. Chemical resistance assessments in 5% sulfuric acid and 5% sodium hydroxide solutions showed strength retention of 95.03% and 91.98%, respectively, outperforming control mixtures by 17% and 13%. SEM examination revealed a dense, cohesive microstructure, reducing shrinkage to 0.01% from 0.15% in the control. These findings underscore the potential of bio-oil and latex–acrylic polymers to improve the performance and sustainability of CAM mixtures, making them well suited for resilient, rapid-setting infrastructure applications.

## 1. Introduction

CAM is widely used in infrastructure projects, particularly for railways [1], bridge expansion joints, and road surfaces, due to its ability to combine the load-bearing strength of cement with the flexibility of asphalt [2]. However, despite these advantages, CAM mixtures face significant challenges. The conventional formulation, which relies heavily on cement, often results in long setting times, high shrinkage, and poor durability under harsh environmental conditions [3,4]. These issues become especially problematic in applications that demand fast installation and high chemical resistance, such as bridge joints in tropical climates characterized by heavy rainfall and temperature fluctuations [5]. Current formulations that aim to improve flexibility or adhesion often compromise on setting time, raising the need for new material innovations to balance both performance and speed.

The use of bio-oil as a sustainable modifier in asphalt mixtures has gained attention as an environmentally friendly alternative to traditional petrochemical-based additives. Bio-oil, derived from biomass sources such as agricultural waste, forestry residues, and even waste cooking oils, is introduced into asphalt mixtures primarily to enhance workability, reduce emissions [6], and improve overall sustainability [7,8]. By partially replacing petroleum-based asphalt binders with bio-oil, research has demonstrated reductions in greenhouse gas emissions associated with asphalt production [9,10]. For example, bio-oil additives have been shown to enhance flexibility and reduce the stiffness of asphalt mixtures, making them particularly suitable for flexible pavement applications where thermal and load-bearing stresses are common [11]. However, optimal bio-oil content is essential, as excessive amounts can disrupt the cohesive structure of asphalt mixtures, leading to potential reductions in mechanical strength and durability under traffic loading.

Polymer modification in asphalt mixtures is another well-researched approach for enhancing pavement performance, particularly in terms of durability, flexibility, and resistance to temperature-related degradation [12,13]. Polymers such as styrene-butadiene-styrene (SBS), poly(styrene-butadiene) latex, and poly(methyl methacrylate) (PMMA) acrylics are commonly used additives in asphalt mixtures to improve flexibility, durability, and resistance to temperature-related degradation. Polymer-modified asphalt (PMA) has shown improved resistance to rutting, thermal cracking, and fatigue, making it highly suitable for applications in regions with high-temperature variability and heavy traffic [14,15]. Polymers create an internal network within the asphalt mixture that improves adhesion and cohesion, which not only enhances load-bearing capacity but also reduces moisture susceptibility, a critical factor in long-term pavement durability. Recent studies have shown that PMA formulations offer superior performance compared to conventional asphalt mixtures, achieving extended service life in various infrastructure applications [16,17]. However, due to their higher cost and complex handling requirements, the practical use of polymers in asphalt is often carefully balanced with performance and economic considerations.

The growing emphasis on sustainable construction has prompted researchers to explore alternatives to petrochemical-based asphalt binders and high cement content. Cement production accounts for a significant portion of global CO_2_ emissions, prompting the industry to adopt low-carbon alternatives to meet sustainability goals [18]. One promising solution is the use of bio-oil, a by-product derived from renewable sources, including vegetable oils, agricultural waste, and biomass pyrolysis. Recent studies suggest that bio-oil improves the workability and flowability of cementitious materials while also reducing shrinkage and cracking during curing [19,20]. However, bio-oil’s impact on early-stage strength and chemical resistance in combination with CAM formulations remains underexplored.

Traditional CAM mixtures struggle to meet the dual demands of sustainable construction and performance in extreme environments. High cement content, while necessary for strength, is associated with increased CO_2_ emissions during manufacturing and can contribute to brittle behavior over time, making structures prone to cracking under thermal stress [21,22]. Moreover, the reliance on petrochemical-based asphalt emulsions further contributes to environmental concerns, limiting CAM’s alignment with the sustainable development goals of modern infrastructure projects [23]. In tropical climates, where heavy rainfall, high humidity, and extreme heat create additional challenges, CAM mixtures often fail to maintain long-term stability due to shrinkage, phase separation, or strength loss at high temperatures [24,25]. Current research efforts have not yet found a solution that adequately addresses both environmental sustainability and fast-setting properties.

This study presents a novel CAM formulation that integrates both bio-oil and polymer additives to address the challenges mentioned. By reducing cement content and partially replacing traditional asphalt emulsion with bio-oil, this approach seeks to enhance the environmental profile of CAM. At the same time, polymer modifiers are introduced to improve adhesion, mechanical strength, and flexibility, ensuring that the material can withstand high temperatures and chemical exposure. This combination of bio-oil and polymer additives represents an innovative strategy to develop a fast-setting, eco-friendly CAM that meets the performance requirements for infrastructure applications while aligning with sustainable construction goals.

**Figure 1:**
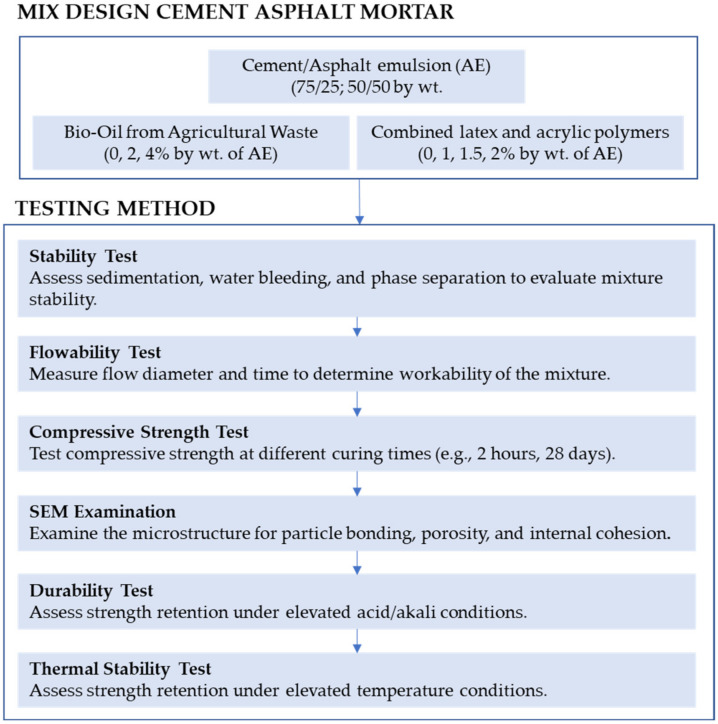
General research flowchart for the development of sustainable cement asphalt mortar.

The experimental phase includes flowability tests to assess the ease of installation, along with compressive strength tests at 2 h and 28 days to monitor early-stage and long-term performance. Shrinkage analysis is conducted to ensure dimensional stability. Additionally, CAM samples are exposed to high-temperature environments (45 °C) and acidic and alkali solutions to evaluate their resistance to environmental stressors. Finally, SEM examination is performed to observe the microstructural changes caused by bio-oil and polymer modifiers, providing insights into the material’s durability and internal bonding characteristics. This study aims to contribute to the body of knowledge by presenting a comprehensive evaluation of bio-oil and polymer-enhanced CAM. The findings will offer practical insights for infrastructure engineers and construction professionals, guiding the design of CAM mixtures that are eco-friendly, durable, and fast-setting. The general research flowchart is presented in Figure 1.

## 2. Materials and Methods

### 2.1. Materials

This section details the specific properties of the materials used in developing bio-oil and polymer-modified CAM. Each material contributes uniquely to the mixture’s performance, ensuring rapid setting, durability, and environmental sustainability.

#### 2.1.1. Cement

The binder used is Type I/II Portland cement, known for its high early strength and resistance to moderate sulfate exposure [26,27]. Its fineness of 3500 cm^2^/g Blaine facilitates rapid hydration, essential for achieving early compressive strength. The specific gravity of 3.15 ensures that the cement integrates well with other components to form a dense mortar matrix. However, cement’s natural tendency to shrink during curing can cause cracks, which is mitigated in this study by incorporating bio-oil and polymer. Lowering cement content also helps reduce the mortar’s carbon footprint, aligning with sustainable construction practices.

#### 2.1.2. Asphalt Emulsion

An anionic asphalt emulsion is used to enhance the flexibility and resistance of the mortar to dynamic stress. This emulsion contains negatively charged asphalt particles with an average particle size of approximately 2 microns, stabilized by an anionic surfactant (sodium lignosulfonate) at a concentration of 0.5% in the aqueous phase [26,27], which interact well with alkaline cement systems, preventing premature coagulation during mixing. With a solid content of 45–50% and viscosity between 500 and 700 cP, the emulsion provides a smooth, homogenous blend, ensuring excellent workability. Asphalt emulsion increases the mortar’s resistance to cracking and thermal expansion, making it ideal for use in infrastructure subjected to temperature changes and vibrations, such as bridges and railways.

#### 2.1.3. Bio-Oil

Bio-oil derived from agricultural waste through pyrolysis is an eco-friendly, renewable additive increasingly used to improve the properties of CAM mixtures. By utilizing agricultural residues, such as crop stalks, husks, and other biomass by-products, bio-oil serves as a sustainable plasticizer that enhances workability while reducing the water demand within CAM [28]. This property is highly beneficial in large-scale infrastructure applications, such as bridge joints, railway beds, and road surfaces, where efficient application, uniform distribution, and minimized void formation are critical for long-term performance and structural integrity.

Precise control over bio-oil concentration is essential, as higher levels can compromise the mechanical strength of CAM. At elevated concentrations, bio-oil may interfere with cement hydration, potentially leading to a weaker matrix and reduced compressive strength. In this study, bio-oil was used at 2% and 4% by emulsion weight. This bio-oil, derived from the pyrolysis of agricultural waste at approximately 500 °C under a nitrogen atmosphere, has a water content of 15%, a density of 1.1 g/cm^3^, and a viscosity of 0.15–0.2 Pa·s. Its composition includes oxygenated organic compounds such as phenols, furans, and ketones, which contribute to its effectiveness as a plasticizer and adhesion enhancer. This controlled use of bio-oil ensures that CAM mixtures retain their strength, resistance to environmental stressors, and sustainability goals, making them suitable for high-performance infrastructure applications. 

#### 2.1.4. Polymer Additives

Polymer additives play a pivotal role in enhancing the mechanical and durability properties of CAM mixtures, particularly in infrastructure applications where flexibility, adhesion, and resistance to environmental stressors are essential. In this study, the latex-based polymer used is poly(styrene-butadiene) with an average molecular weight of approximately 150,000 g/mol, and the acrylic polymer is poly(methyl methacrylate) (PMMA) with an average molecular weight of around 120,000 g/mol [29]. These polymers are known to modify the internal structure of CAM mixtures by forming a flexible yet cohesive network within the cement matrix, thus creating stronger internal bonds. This modified structure reduces the likelihood of shrinkage, cracking, and delamination under both thermal and mechanical stresses, making the mixture suitable for high-performance applications such as bridge expansion joints, railways, and pavements. By reinforcing internal cohesion, these polymer additives support the CAM in tolerating dynamic and cyclic loads while also improving durability over time.

One of the primary benefits of adding polymers to CAM is their ability to enhance flexibility and adhesion within the cement matrix. The polymers form a continuous film, effectively binding cement particles and aggregates, which strengthens the mixture and mitigates the risk of cracking. Latex polymers, for instance, contribute notable flexibility to the mortar, enhancing its ability to withstand dynamic and thermal stresses without losing structural integrity [29]. This quality is especially important in large infrastructure projects where the materials must endure thermal expansion, contraction, and repeated load-bearing cycles. Acrylic polymers complement this by further reinforcing the matrix’s adhesion and cohesion, making it less prone to delamination under load. This combined strength and flexibility are crucial in providing CAM with a resilient bond, especially at joints and other connection points where materials are susceptible to wear.

Thermal stability is another critical advantage offered by polymer additives, particularly in regions with extreme or fluctuating temperatures. Latex polymers, with a glass transition temperature (Tg) in the range of 0–5 °C, enhance elasticity, allowing the mortar to expand and contract with temperature changes without fracturing. This thermal flexibility minimizes the likelihood of microcracking, thereby reducing maintenance needs and extending the lifespan of CAM in infrastructure applications. Acrylic polymers contribute additional stability, bolstering the mixture’s resistance to temperature-induced stress. The combination of these polymers supports CAM in maintaining both structural integrity and elasticity across a range of temperatures, which is essential for outdoor applications exposed to seasonal and daily temperature fluctuations.

In this study, the polymer additive proportion is optimized at a total of 1.5% by weight of the CAM mixture. This consists of a balanced combination of 1.0% latex polymer and 0.5% acrylic polymer. This ratio is selected to leverage the flexibility and elasticity offered by latex polymers, while the acrylic polymer enhances adhesion, cohesion, and chemical resistance. The balanced 1:0.5 ratio of latex to acrylic ensures that the CAM mixture achieves optimal durability and resilience, effectively supporting infrastructure exposed to dynamic loads and environmental stressors, without compromising the mixture’s workability or dimensional stability. 

#### 2.1.5. Fine Sand

Fine sand, with particles sized between 0.1 and 2 mm, provides structural support and reduces shrinkage. The angular grains improve bonding with the cement matrix, increasing the mortar’s mechanical strength. Sand also acts as a filler, reducing voids within the mixture and minimizing shrinkage during curing.

#### 2.1.6. Water

The water used is potable tap water with a pH value of 6.5–8.5 to ensure compatibility with cementitious systems. A water-to-cement ratio of 0.4 is maintained to balance workability and strength. In this study, the water-to-cement (w/c) ratio of 0.4 refers specifically to the added water content and does not include the water present in the emulsified asphalt. The emulsified asphalt contains a certain amount of water that does not directly participate in the hydration of cement but contributes to the overall workability and consistency of the mixture. Similarly, the bio-oil used in the mixtures contains a water content of approximately 15%, which, although not actively involved in the cement hydration process, plays a role in enhancing the workability and distribution of the components within the CAM matrix. This additional moisture from bio-oil helps in reducing the reliance on external water addition, thereby supporting uniformity and consistency during mixing.

#### 2.1.7. Superplasticizer

The polycarboxylate-based superplasticizer used in this study is a polycarboxylate ether polymer with an average molecular weight of approximately 50,000 g/mol, consisting of a polycarboxylate backbone with polyethylene glycol side chains. It was added at a concentration of 0.3% by weight of the cementitious material. 

#### 2.1.8. Defoaming Agent

A silicone-based defoaming agent, specifically polydimethylsiloxane with a kinematic viscosity of approximately 100 cSt, was used to minimize air entrainment in the CAM mixture. Based on suggestions from previous research [26], the agent was added at 0.1% of the total weight to ensure consistent mechanical properties by effectively eliminating air voids that could weaken the structure. The overview of the properties of the featured material used in this research is presented in Table 1. 

### 2.2. Mix Design

The mix design for this study focuses on balancing workability, strength, durability, and sustainability by varying the proportions of cement, asphalt emulsion, bio-oil, and polymer additives. Seven distinct mixtures were prepared to systematically investigate the impact of these variables on the performance of CAM. The control mixture (M1) contains 100% cement without any bio-oil or polymer additives, serving as a benchmark for evaluating the modifications in the other formulations. Cement content in the experimental mixtures is reduced incrementally, ranging from 75% to 25%, with corresponding increases in asphalt emulsion to explore the effect of cement reduction on compressive strength and shrinkage [30]. In addition, bio-oil is incorporated at two levels, 5% and 10%, to assess its role in improving workability and sustainability. Selected mixtures also contain 2–3% polymer additives to enhance adhesion, flexibility, and chemical resistance. The mix design of the CAM mixture is presented in Table 2.

The detailed proportions of each material used in the study are provided in Table 2. All formulations maintain consistent levels of water (40%), sand (50%), superplasticizer (2%), and defoaming agent (0.1%) to ensure comparability across the tests. By systematically varying cement, bio-oil, and polymer content, the study aims to determine the most effective formulation for balancing rapid setting time with long-term durability, while also considering environmental impact through the use of bio-oil.

The mixing procedure involves a two-stage process designed to ensure uniformity and eliminate phase separation. The dry mixing stage begins with cement and fine sand, which are blended for two minutes at 350 revolutions per minute (RPM) to achieve a consistent distribution of the dry components. In the wet mixing stage, asphalt emulsion, bio-oil, water, polymer additives, superplasticizers, and defoaming agents are gradually added to the dry blend. The wet mixing continues for seven minutes at the same speed, ensuring the complete incorporation of all ingredients. Special care is taken during the mixing process to avoid air entrapment, which could weaken the mechanical structure of the mortar. The defoaming agent is included in small quantities to prevent the formation of air bubbles and ensure that the final material exhibits consistent mechanical properties.

The mix design explores the interaction between cement reduction, bio-oil addition, and polymer modification to determine their combined effects on flowability, compressive strength, shrinkage, and chemical resistance. Varying the cement-to-emulsion ratio allows the study to assess how cement reduction impacts mechanical properties while improving flexibility. The addition of bio-oil serves to improve the workability of the mortar and reduce its environmental footprint by minimizing the reliance on high cement content. The polymer additives enhance the mortar’s ability to withstand thermal expansion and chemical degradation, critical for applications in infrastructure exposed to extreme weather and environmental stressors. This careful mix design provides a robust framework for evaluating the optimal balance of performance and sustainability in CAM formulations.

### 2.3. Experimental Procedures

The following experimental procedures were meticulously designed to ensure reproducibility and reliability of results under controlled environmental conditions. Tests were conducted to evaluate the workability, strength, dimensional stability, thermal durability, and chemical resistance of the bio-oil and polymer-enhanced CAM mixtures. Each test followed ASTM standards, and specific testing conditions, including temperature, humidity, curing time, sample dimensions, and loading rates, were applied to simulate real-world infrastructure scenarios.

#### 2.3.1. Mixing and Sample Preparation

The CAM mixtures were prepared using a mechanical mixer operating at 350 RPM. Each batch was mixed for a total of 9 min: 2 min for dry mixing (cement and sand) and 7 min for wet mixing (asphalt emulsion, bio-oil, polymer additives, water, superplasticizer, and defoaming agent). The freshly mixed mortar was poured into standard molds with dimensions specific to each test:

- Compressive strength: 50 mm in diameter × 100 mm in height.
- Shrinkage and flowability: Prismatic molds (25 mm × 25 mm × 285 mm).
- Chemical resistance and thermal tests: Cylindrical molds (50 mm diameter, 100 mm height).

**Figure 2:**
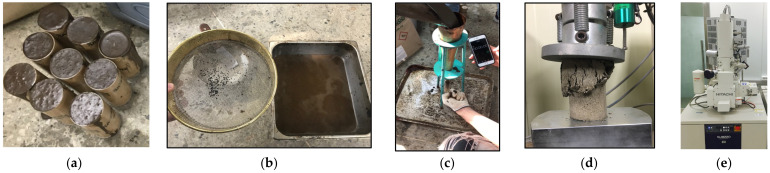
Sample preparation and testing procedures: (**a**) sample preparation, (**b**) mixing stability test, (**c**) flowability test, (**d**) unconfined compressive strength (UCS) test, (**e**) SEM examination.

The samples were compacted using a vibrating table for 30 s to eliminate air voids. After molding, the specimens were covered with plastic sheets and demolded after 24 h, followed by standard curing at 23 ± 2 °C and 50 ± 5% relative humidity for 28 days, depending on the test (see Figure 2a). 

Each mixture undergoes controlled mixing to achieve uniformity, and the stability is then evaluated based on visible phase separation and measurements of sedimentation (see Figure 2b) and water bleeding. This process helps determine the ideal mix for mechanical performance and durability.

#### 2.3.2. Flowability Test

The flowability of the mortar was assessed following ASTM C230 [31]. A conical mold was filled with mortar and placed on the flow plate (see Figure 2c). The flow time, defined as the time taken for the mortar to reach 250 mm in diameter, was also recorded. The test was repeated three times for each mixture to ensure consistency, with environmental conditions maintained at 23 ± 2 °C and 50 ± 5% relative humidity.

#### 2.3.3. Compressive Strength

The compressive strength tests were performed according to ASTM C39 for early-stage strength (2-h) and for 28-day strength [32]. The mortar cubes (50 mm in diameter × 100 mm in height) were loaded using a universal testing machine at a loading rate of 1 kN/s until failure (see Figure 2d). The tests were conducted under ambient conditions of 23 ± 2 °C and 50 ± 5% relative humidity. Each mixture was tested in triplicate, and the average strength was recorded for analysis. For 2 h compressive strength, samples were cured in a temperature-controlled water bath at 20 ± 1 °C. For the 28-day strength, samples were stored in a curing chamber at 23 ± 2 °C and 98% relative humidity.

#### 2.3.4. Shrinkage Analysis

Shrinkage tests followed ASTM C157 to evaluate dimensional stability over time [33]. Prismatic samples (25 mm × 25 mm × 285 mm) were cast and measured using a digital length comparator. After demolding, the specimens were cured in a chamber at 50 ± 5% humidity and 23 ± 2 °C. The length change of each sample was monitored to assess the risk of shrinkage-induced cracking. Three specimens per mixture were tested, and the average length change was reported as the percentage shrinkage.

#### 2.3.5. High-Temperature Resistance Test

To simulate tropical climate conditions, high-temperature resistance tests were conducted at 45 °C following ASTM C39 [33]. After 7 days of standard curing, the cylindrical samples (50 mm diameter, 100 mm height) were placed in a temperature-controlled oven at 45 °C for 24 h. The compressive strength was measured after exposure, and the percentage reduction in strength was calculated relative to the samples tested at room temperature. Three specimens from each mixture were tested to ensure statistical reliability.

#### 2.3.6. Chemical Resistance Test

The chemical resistance of the CAM mixtures was evaluated through immersion tests based on ASTM C267 [33]. For this test, cylindrical samples with dimensions of 100 mm in diameter and 200 mm in height were fully submerged in two separate solutions: 5% sulfuric acid (H_2_SO_4_) and 5% sodium hydroxide (NaOH). The samples remained in these solutions for 14 days, during which the pH levels of each solution were periodically monitored and adjusted as needed to ensure consistent acidity or alkalinity throughout the test period. This approach allowed us to assess the durability of the CAM mixtures under prolonged exposure to both acidic and alkaline environments.

At the end of the immersion period, the samples were washed and allowed to dry for 24 h before their compressive strength was measured. The percentage loss in strength was calculated to determine the resistance of each mixture to chemical degradation. Three replicates per solution were tested.

#### 2.3.7. SEM Examination

SEM was used to examine the internal microstructure of the CAM mixtures (see Figure 2e). Small fragments from the cured mortar samples were mounted on stubs, coated with gold, and observed under an SEM with a magnification range of 1000× to 5000×. The test focused on examining the distribution and interaction of cement particles, asphalt emulsion, bio-oil, and polymer additives within the microstructure, as observed through SEM. This approach provided insights into the cohesion and spatial arrangement of these components within the CAM matrix. SEM imaging provided insights into how the microstructure affected the mechanical and chemical properties of the mixtures.

## 3. Results and Discussions

### 3.1. Mixing Stability Test Results

**Figure 3:**
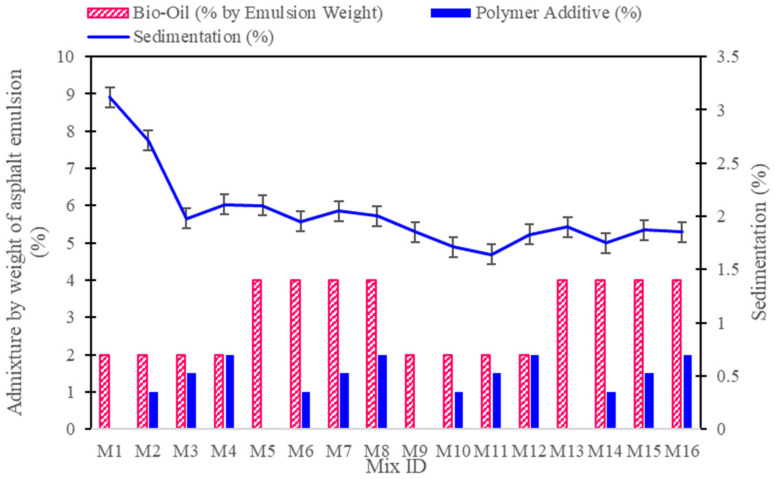
Results of mixing stability test for CAM mixtures with various cement-to-asphalt ratios and additive levels.

The mixing stability of CAM mixtures, as measured by sedimentation percentage, illustrates the effects of different cement-to-asphalt ratios, bio-oil concentrations, and polymer additive levels on the uniformity and phase stability of the mixtures. Lower sedimentation percentages indicate improved stability, with reduced particle settlement and phase separation. As shown in Figure 3, the results demonstrate that mixtures with a 50:50 cement-to-asphalt ratio generally exhibit better stability (lower sedimentation) than those with a 75:25 ratio, suggesting that a higher asphalt emulsion content enhances the mixture’s lubrication, reducing internal friction and sedimentation.

In mixtures with a 75:25 cement-to-asphalt ratio and 2% bio-oil, the addition of polymers enhances the cohesion and flexibility of the CAM mixtures, contributing to improved mechanical properties and durability. For instance, M1 (0% polymer) shows a sedimentation percentage of 3.12%, while increasing the polymer to 1% in M2 reduces sedimentation to 2.71%. The stability improves further in M3 with 1.5% polymer, reaching a sedimentation of 1.98%, indicating that polymer additives enhance cohesion by binding particles and minimizing separation. However, at higher polymer levels, such as in M4 (2% polymer), the sedimentation increases slightly to 2.11%, suggesting that excessive polymer may increase viscosity, thus limiting flow and causing minor sedimentation.

For mixtures with 4% bio-oil and a 75:25 cement-to-asphalt ratio, bio-oil’s plasticizing effect initially reduces sedimentation, as seen in M5 (2.1% sedimentation, no polymer). Adding 1% polymer in M6 further reduces sedimentation to 1.95%, indicating that a combination of bio-oil and polymer creates a well-balanced, stable mixture. However, with higher polymer levels, the sedimentation begins to stabilize around 2% (e.g., M7 and M8), implying that the stability effect of bio-oil is maximized when combined with moderate polymer concentrations. Excessive polymer does not significantly enhance stability at this level of bio-oil.

In the 50:50 cement-to-asphalt mixtures, lower sedimentation percentages were observed across all bio-oil and polymer levels, emphasizing the stabilizing effect of increased asphalt content. For example, M9 (2% bio-oil, no polymer) shows a sedimentation of 1.85%, which is further reduced to 1.64% in M11 with 1.5% polymer. This suggests that the asphalt emulsion provides a lubricating effect, reducing particle settlement and enhancing cohesion within the matrix. With 4% bio-oil, the mixtures remain stable, with M13 (no polymer) showing 1.9% sedimentation and M15 (1.5% polymer) achieving a similar value of 1.87%. The consistency in sedimentation values with higher asphalt and bio-oil content underscores the importance of asphalt emulsion in maintaining homogeneity and reducing phase separation.

Overall, these findings suggest that both polymer and bio-oil additives contribute to improving mixing stability, with an optimal balance achieved by moderate polymer concentrations (1–1.5%) and bio-oil levels around 2%, particularly in 50:50 cement-to-asphalt mixtures. Higher bio-oil levels (4%) can be effectively stabilized with moderate polymer additions, but excessive polymer may diminish flowability without significantly reducing sedimentation. 

These results demonstrate that polymer reinforcement is essential in maintaining mixing stability, especially at higher bio-oil concentrations. The findings also confirm that the 50:50 cement-to-asphalt ratio provides better homogeneity than the 75:25 ratio, further emphasizing the importance of optimizing cement reduction and additive content for improved performance.

### 3.2. Flowability Test Results

**Figure 4:**
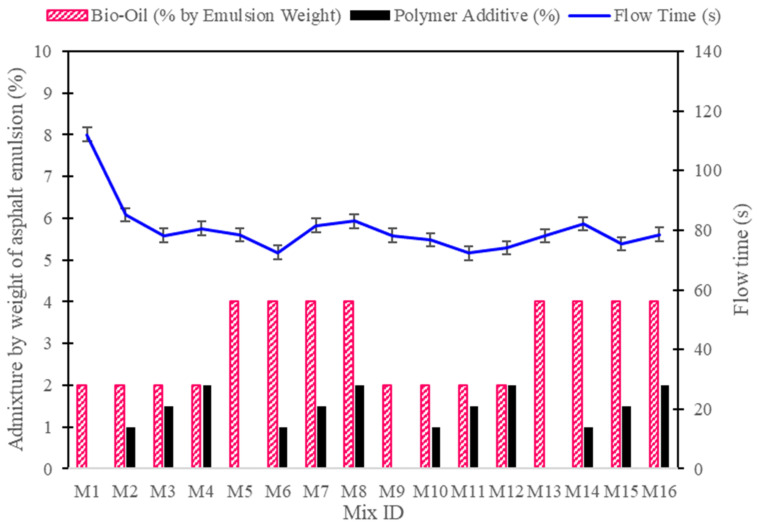
Flowability test results of CAM mixtures with varying cement, bio-oil, and polymer concentrations.

The flowability test results, as indicated by flow time, highlight the impact of cement-to-asphalt ratios, bio-oil concentrations, and polymer additives on the workability of CAM mixtures as shown in Figure 4. Lower flow times generally indicate better flowability, which is beneficial for ease of application. Mixtures with a 75% cement content and 2% bio-oil displayed improved flowability as polymer additives were introduced. For instance, M1 (75% cement, 2% bio-oil, 0% polymer) recorded a flow time of 112.1 s, showing poor flowability. However, adding 1% polymer in M2 reduced the flow time significantly to 85.12 s, while M3 (1.5% polymer) achieved an optimal flow time of 78.2 s. This trend indicates that a moderate level of polymer improves workability by enhancing internal cohesion without overly increasing viscosity. Increasing the polymer content to 2% (M4) slightly increased flow time to 80.56 s, suggesting that too much polymer might begin to reduce flowability by increasing mixture viscosity.

For mixtures with 4% bio-oil and a 75:25 cement-to-asphalt ratio, the trend is slightly different. The introduction of bio-oil without polymer in M5 improved flow time to 78.5 s, indicating that bio-oil acts as a plasticizer, enhancing the mix’s fluidity. However, adding polymer to these high-bio-oil mixtures had a varied effect; M6 (1% polymer) achieved the lowest flow time of 72.5 s, indicating optimal workability, while M7 and M8, with 1.5% and 2% polymer, respectively, showed increased flow times of 81.64 and 83.1 s. This suggests that, in high-bio-oil mixtures, a small amount of polymer (around 1%) enhances flowability, but higher polymer concentrations increase viscosity, diminishing the beneficial effects of bio-oil on flowability.

In the 50:50 cement-to-asphalt ratio mixtures, a similar pattern is observed. M9, the control with 2% bio-oil and no polymer, had a flow time of 78.2 s, indicating better flowability than its 75% cement counterpart (M1). Adding polymer to these mixtures further improved flow, with M11 (1.5% polymer) achieving the lowest flow time of 72.3 s, suggesting that the combination of 50% asphalt emulsion and moderate polymer content enhances lubrication within the mixture, reducing internal friction. However, at 4% bio-oil, the flow time slightly increased as polymer concentration rose, with M14 (1% polymer) recording 82.1 s and M16 (2% polymer) at 78.56 s. This implies that, while 50:50 mixtures generally maintain good flowability, excessive polymer in high-bio-oil formulations can reduce flowability by increasing matrix viscosity.

In summary, the flowability results indicate that both bio-oil and polymer additives play critical roles in adjusting workability. Moderate levels of polymer (around 1–1.5%) combined with 2% bio-oil generally achieve optimal flowability, especially in mixtures with higher asphalt content (50% asphalt emulsion). However, excessive polymer concentrations can counteract the beneficial effects of bio-oil, leading to increased viscosity and reduced flowability. 

### 3.3. Compressive Strength Test Results

**Figure 5:**
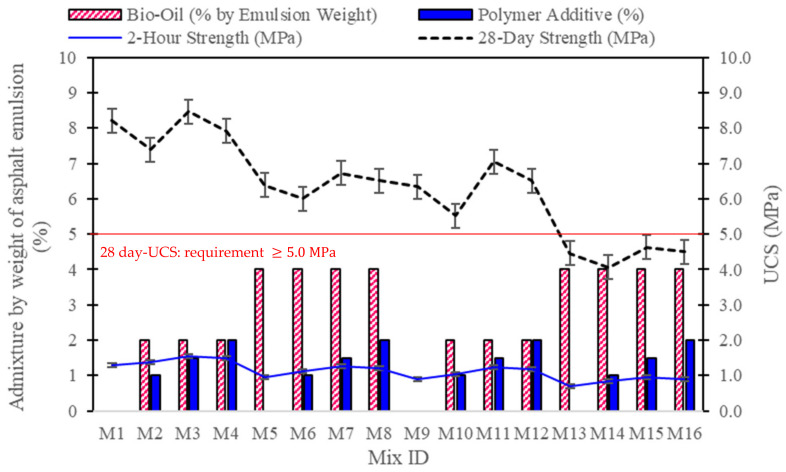
Compressive strength test results for CAM mixtures at 2-h and 28-day intervals.

The unconfined compressive strength (UCS) results at both 2 h and 28 days reveal the effects of varying cement-to-asphalt ratios, bio-oil concentrations, and polymer additive levels on the mechanical performance of CAM mixtures (see Figure 5). In general, mixtures with a 75% cement content show higher 28-day strengths compared to those with a 50% cement content, underscoring the role of cement in providing long-term structural integrity. For instance, the control mixture M1 (75% cement, 0% additives) recorded a 28-day strength of 8.2 MPa, whereas M9, the 50% cement control, achieved only 6.3 MPa. The addition of 2% bio-oil and polymer additives (1–2%) in the 75% cement mixtures generally enhanced both 2 h and 28-day strengths, with M3 (2% bio-oil, 1.5% polymer) achieving the highest values of 1.6 MPa at 2 h and 8.5 MPa at 28 days. This suggests that moderate bio-oil and polymer additions can improve early strength while maintaining long-term performance, likely due to improved internal cohesion and reduced porosity provided by the polymer coating on asphalt particles.

In contrast, increasing the bio-oil concentration to 4% in the 75% cement mixtures resulted in lower compressive strengths. For example, M5 (4% bio-oil, no polymer) recorded a 2 h strength of 1.0 MPa and a 28-day strength of 6.4 MPa, indicating that high bio-oil levels without polymer reinforcement reduce strength by overly plasticizing the matrix. However, adding polymer (1–2%) to the 4% bio-oil mixtures (M6–M8) partially mitigated this effect, as evidenced by M7 (1.5% polymer), which reached a 28-day strength of 6.7 MPa, though still lower than mixtures with 2% bio-oil. This highlights the need for balanced bio-oil and polymer concentrations to maintain compressive strength.

The 50:50 cement-to-asphalt mixtures displayed generally lower UCS values compared to their 75% cement counterparts, particularly at higher bio-oil concentrations. For instance, M13 (4% bio-oil, no polymer) recorded the lowest strengths, with 0.7 MPa at 2 h and 4.5 MPa at 28 days, emphasizing that reduced cement content combined with high bio-oil concentrations weakens the matrix. Among the 50:50 mixtures, M11 (2% bio-oil, 1.5% polymer) showed the best performance, achieving a 2 h strength of 1.2 MPa and a 28-day strength of 7.0 MPa. This suggests that a lower bio-oil concentration, balanced with polymer reinforcement, is more suitable for mixtures with reduced cement content. Overall, while bio-oil enhances workability and flexibility, excessive amounts reduce compressive strength, especially in low-cement formulations. A moderate level of bio-oil (2%) and polymer (1.5%) provides an optimal balance for maximizing strength in both high- and low-cement CAM mixtures.

### 3.4. Expansion Characteristics Analysis

The analysis of the expansion characteristics of the CAM mixtures is pivotal in assessing their dimensional stability under varying environmental conditions. This study reveals that the addition of bio-oil and polymer additives significantly impacts the shrinkage behavior of the mixtures, which is crucial for applications where precise dimensions must be maintained.

**Figure 6:**
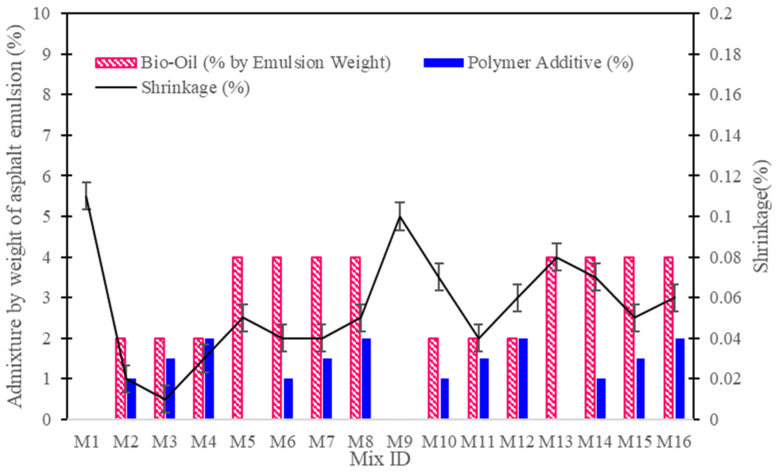
Shrinkage test results showing dimensional stability of CAM mixtures with different additive combinations.

As presented in Figure 6, The control mixture (M1), which contains 100% cement, exhibited notable shrinkage of −0.11%. This high shrinkage rate indicates a potential risk of cracking and dimensional instability, especially in environments characterized by temperature fluctuations and moisture variations. Conversely, the incorporation of 2% bio-oil and polymer additives drastically improved the shrinkage characteristics of the mixtures. For instance, M3 (75:25 ratio, 2% bio-oil, 1.5% polymer) demonstrated a remarkable reduction in shrinkage to −0.01%. This significant improvement can be attributed to the ability of the polymers to enhance the internal structure, thereby minimizing stress concentrations and improving flexibility.

Mixtures with 4% bio-oil, while still maintaining reasonable performance, displayed slightly increased shrinkage. M7 (75:25 ratio, 4% bio-oil, 1.5% polymer) showed a shrinkage value of −0.04%, indicating that higher bio-oil concentrations can lead to a less stable microstructure. This is likely due to the plasticizing effect of excess bio-oil, which can hinder the hydration process and make the material more susceptible to dimensional changes. This observation aligns with previous research, which suggests that while bio-oil enhances workability, excessive amounts can negatively impact the dimensional stability of cementitious materials [21].

For the 50:50 cement-to-asphalt ratio mixtures, the trend of reduced shrinkage with polymer addition persisted. M11 (50:50 ratio, 2% bio-oil, 1.5% polymer) recorded shrinkage of −0.04%, demonstrating that the polymers continue to provide effective mitigation against shrinkage even in mixtures with lower cement content. This finding supports the notion that polymer additives can significantly enhance the dimensional stability of CAM mixtures, making them more suitable for infrastructure applications requiring precise dimensional control.

### 3.5. Acid and Alkali Resistance

**Figure 7:**
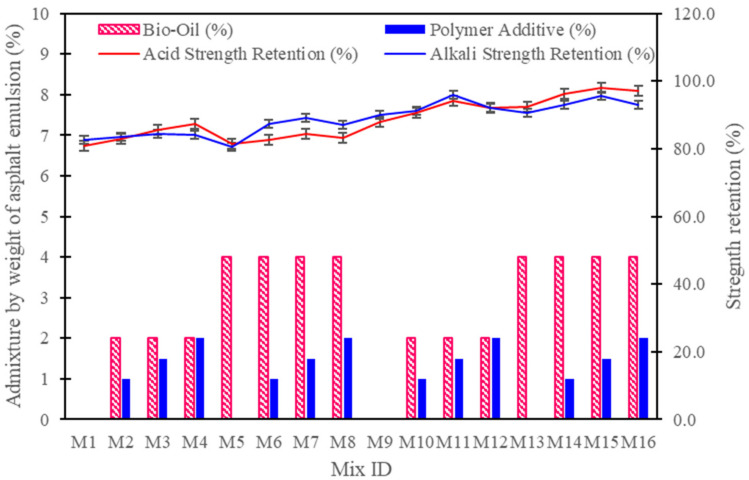
Acid and alkali resistance test results for CAM mixtures with varying additive levels.

The acid and alkali strength retention data reveal significant insights into how varying cement-to-asphalt ratios, bio-oil concentrations, and polymer additives affect the chemical durability of CAM mixtures as shown in Figure 7. Generally, mixtures with a 50:50 cement-to-asphalt ratio perform better in both acid and alkali retention than those with a 75:25 ratio. For example, the control mixture M1 (75% cement, 0% bio-oil, 0% polymer) showed acid and alkali retention rates of 81.0% and 82.5%, respectively, whereas the control mixture M9 with a 50:50 ratio (no additives) exhibited higher retention rates of 87.8% and 90.2%. This improvement can be attributed to the increased asphalt emulsion content, which enhances flexibility and reduces brittleness, creating a matrix that better withstands chemical degradation.

The addition of bio-oil and polymer has a further enhancing effect, but the response varies depending on the concentration and type of additives. In the 75:25 cement-to-asphalt ratio mixtures, adding 2% bio-oil along with polymer (1–2%) progressively improved acid and alkali retention. For instance, M2, with 1% polymer and 2% bio-oil, achieved 83.0% acid retention and 83.5% alkali retention, while M3 (with 1.5% polymer) reached 85.6% acid retention and 84.4% alkali retention. This trend continued with M4, which had 2% polymer and achieved 87.3% acid retention and 84.1% alkali retention. These results suggest that polymer additives effectively strengthen the CAM matrix against acid and alkali attacks by enhancing the cohesion and coating of asphalt particles, which improves the mixture’s overall integrity under chemical exposure. However, increasing bio-oil to 4% without polymer additives, as in M5, led to reduced retention rates of 81.5% and 80.7%. This indicates that while bio-oil alone can improve workability and flexibility, high concentrations without polymer support may compromise chemical resistance by weakening the cohesive structure of the matrix through excessive plasticization.

In contrast, the 50:50 cement-to-asphalt ratio mixtures demonstrate higher acid and alkali retention, especially when bio-oil and polymer are combined at optimal levels. Mixtures M10–M12, with 2% bio-oil and 1–2% polymer, achieved acid retention rates of 90.7%, 94.1%, and 92.0% and alkali retention rates of 91.2%, 96.0%, and 92.1%, respectively. Among these, M11 (with 1.5% polymer) showed the best balance between acid and alkali resistance, suggesting that a moderate polymer level is ideal for durability in chemically aggressive environments. When bio-oil concentration was increased to 4% with varying polymer levels, the impact became more pronounced. Mixture M14 (50:50 ratio, 4% bio-oil, 1% polymer) achieved 96.3% acid retention and 93.1% alkali retention, while M15 (4% bio-oil, 1.5% polymer) exhibited the highest chemical durability, with 98.0% acid retention and 95.5% alkali retention. These results indicate a synergistic effect where higher bio-oil content, when balanced by sufficient polymer, enhances the flexibility and cohesion of the matrix, thereby improving chemical resistance. This is likely because the polymer aids in coating the asphalt emulsion, forming a stronger, more resistant internal structure that is less susceptible to degradation under acidic or alkaline conditions.

Comparatively, M16, with 4% bio-oil and 2% polymer, had slightly lower retention rates (97.0% acid and 93.0% alkali) than M15. This suggests that while polymers improve strength retention, an excessive polymer concentration (beyond 1.5%) might lead to diminishing returns, potentially by encapsulating cement particles and reducing the matrix’s bonding efficiency. Overall, the data align with previous studies, which indicate that bio-based plasticizers like bio-oil enhance the workability and flexibility of CAM mixtures, while polymer additives provide essential cohesion and improve the resistance to chemical attacks. The best-performing mixture, M15, demonstrates that a 50:50 cement-to-asphalt ratio with 4% bio-oil and 1.5% polymer strikes an optimal balance, achieving high durability without compromising structural integrity. These findings underscore the importance of balancing bio-oil’s plasticizing effects with polymer’s cohesive benefits for chemically resilient CAM formulations.

### 3.6. Thermal Stability Test Results

**Figure 8:**
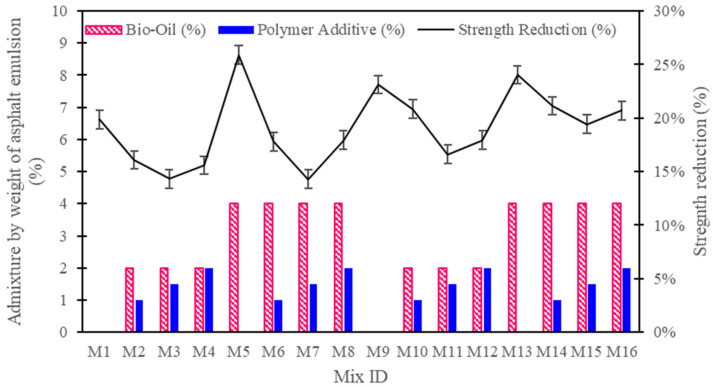
Thermal stability test results: strength retention of CAM mixtures after exposure to elevated temperatures.

The thermal stability test results reveal key insights into how different CAM mixtures, particularly those modified with bio-oil and polymer additives, behave under elevated temperatures (see Figure 8). The general trend observed across the mixtures indicates that polymer-modified CAMs exhibit significantly better thermal stability than mixtures without polymer additives, retaining a higher percentage of their original compressive strength after exposure to 45 °C for 24 h. Notably, the mixtures containing 1.5% polymer additives (M3, M7, and M11) demonstrated the highest strength retention, with strength reductions of 14.31%, 14.30%, and 16.61%, respectively. These results suggest that the addition of polymers helps create a more cohesive internal structure, improving the material’s resistance to microcracking and phase changes that might occur during thermal exposure. This finding aligns with previous research indicating that polymers form strong, flexible networks within cementitious materials, which helps mitigate the effects of thermal stress [34].

In contrast, the control mixtures without polymer additives, such as M1 and M9, exhibited higher strength reductions of 19.88% and 23.13%, respectively. The absence of polymers in these formulations likely led to weaker internal bonding, making the material more susceptible to thermal-induced degradation. Similarly, the mixtures with higher bio-oil content but no polymer reinforcement, such as M5 and M13, showed even greater strength losses of 25.89% and 24.03%, respectively. These results can be attributed to the plasticizing effect of bio-oil, which, while improving workability, appears to compromise the material’s ability to maintain structural integrity under high temperatures, especially in the absence of polymers. Bio-oil tends to interfere with the hydration process of cement, potentially leading to a less dense microstructure that is more vulnerable to thermal fluctuations.

Comparing the performance of the 75:25 and 50:50 cement-to-asphalt ratio mixtures, the 75:25 mixtures generally outperformed their 50:50 counterparts in terms of thermal stability. For example, M3 (75:25 ratio, 2% bio-oil, 1.5% polymer) experienced a strength reduction of only 14.31%, while M11 (50:50 ratio, 2% bio-oil, 1.5% polymer) had a reduction of 16.61%. This difference highlights the critical role that cement content plays in maintaining the strength of CAM mixtures at elevated temperatures. The higher cement content in the 75:25 mixtures leads to a more robust hydration process, resulting in a denser and more thermally stable matrix. This finding is consistent with earlier studies that suggest a higher cement-to-asphalt ratio improves the thermal resistance of cementitious composites due to the formation of a more continuous cementitious matrix [21].

It is also evident that the inclusion of polymer additives is essential in improving the thermal stability of CAM mixtures, particularly at higher bio-oil concentrations. Mixtures M7 and M15, which contained 4% bio-oil and 1.5% polymer, retained 14.30% and 19.44% of their strength, respectively, demonstrating that the polymer additives effectively counterbalance the weakening effects of bio-oil. Without polymer reinforcement, as seen in M5 and M13, the thermal stability deteriorates, indicating that the combination of bio-oil and polymers must be carefully balanced to achieve optimal performance.

The findings suggest that a 75:25 cement-to-asphalt ratio, combined with 1.5% polymer and 2% bio-oil, offers the best balance between thermal stability and sustainability, retaining strength while minimizing environmental impact. Future research could explore the long-term durability of these mixtures in real-world high-temperature conditions to further validate these findings.

### 3.7. SEM Test Results

The SEM examination was carried out to provide a qualitative understanding of the microstructural characteristics of CAM mixtures modified with bio-oil and polymer additives. The analysis focused on observing general trends in internal cohesion, porosity, and asphalt emulsion (AE) coating without drawing definitive conclusions due to the limitations in image resolution.

**Figure 9:**
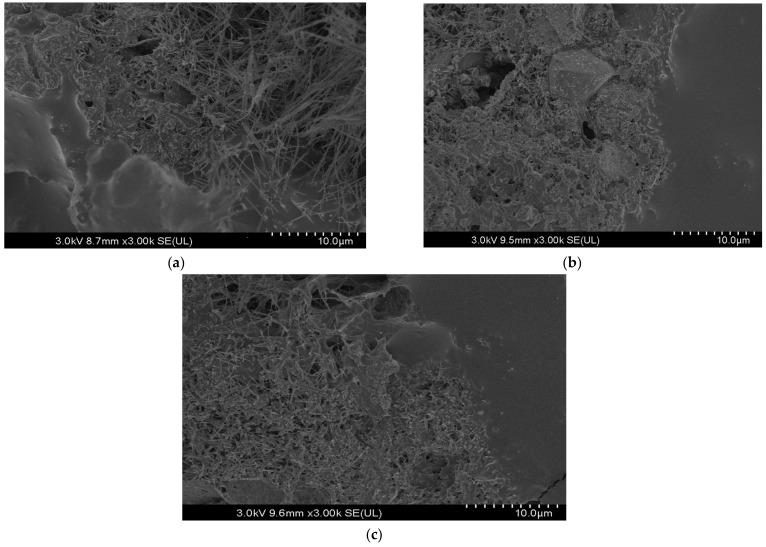
SEM microstructural analysis of CAM mixtures: (**a**) control mixture (no bio-oil or polymer), (**b**) mixture with 4% bio-oil, (**c**) optimized mixture with 1.5% polymer and 2% bio-oil.

Figure 9a represents the control mixture (M1), which does not include bio-oil or polymer additives. The SEM image reveals a dense yet brittle structure with visible hydration products characteristic of cement hydration. However, there is no clear evidence of a coating around the asphalt particles, suggesting limited cohesion and weak internal bonding. This may contribute to dimensional instability and mechanical vulnerabilities observed in this mixture.

Figure 9b illustrates the mixture containing 4% bio-oil without polymer additives. The image suggests a more dispersed particle arrangement compared to the control, with slightly reduced porosity. However, the coating of the asphalt emulsion appears inconsistent, and voids are still noticeable. These observations point to the potential of bio-oil in enhancing particle distribution but also indicate the need for additional reinforcement, such as polymers, to improve cohesion and stability.

Figure 9c shows the microstructure of the optimized mixture containing 1.5% polymer and 2% bio-oil. While voids are still present, the SEM image indicates a more developed C-S-H system forming a denser matrix. Additionally, the asphalt emulsion appears to be better coated, with the polymer contributing to a more cohesive and uniform microstructure. This improved interaction between the components suggests better internal bonding and reduced porosity compared to the other mixtures.

While these observations provide insights into the potential benefits of bio-oil and polymer additives, the limitations in image quality restrict the scope of definitive conclusions. Further studies with higher-resolution imaging and complementary quantitative methods are recommended to confirm the microstructural trends identified.

### 3.8. Discussions

Based on the findings of this research, the modified structure of the CAM mixture, achieved through the incorporation of polymer additives, demonstrated strong durability under both thermal and mechanical stresses. This enhancement is particularly beneficial for high-performance applications such as bridge expansion joints, railways, and pavements, where materials are subjected to dynamic and cyclic loading.

The polymers effectively reinforced internal cohesion within the cement matrix, creating a flexible yet robust network that could withstand environmental and operational stressors. This improved structural integrity translated to enhanced durability over time, ensuring the CAM mixtures maintained their performance under prolonged exposure to varying conditions. These findings underscore the importance of polymer modifications in optimizing the mechanical and thermal stability of CAM for infrastructure applications.

## 4. Conclusions

This study successfully developed a sustainable CAM formulation incorporating bio-oil derived from agricultural waste and polymer additives, designed to meet the dual demands of environmental sustainability and high-performance requirements in infrastructure materials. The integration of bio-oil and polymers in the CAM mixture addresses key limitations found in conventional formulations, such as limited workability, high shrinkage, and reduced durability under extreme conditions.

The findings demonstrate that bio-oil and polymer additives offer distinct advantages: bio-oil functions as a plasticizer, enhancing flowability and reducing water demand, while polymers strengthen internal cohesion, reduce sedimentation, and improve the mixture’s resistance to thermal and chemical stressors. Specifically, an optimal combination of 2% bio-oil and 1.5% polymer in a 75:25 cement-to-asphalt ratio produced the most balanced properties, achieving reduced shrinkage, improved compressive strength, and increased dimensional stability. This formulation retained 85% of its compressive strength under thermal exposure at 45 °C and showed over 95% strength retention in acidic and alkaline environments, underscoring its suitability for challenging conditions.

Additionally, the microstructural analysis confirmed a dense and cohesive matrix with minimal voids, providing physical integrity and resistance to environmental degradation. These improvements suggest that the optimized CAM formulation is well-suited for applications in regions with fluctuating temperatures, high humidity, or frequent exposure to aggressive chemicals, such as bridge joints, railways, and pavements in tropical climates.

Future research should focus on field trials to validate the long-term performance of these formulations and explore alternative bio-oil sources to further optimize the environmental benefits. The insights gained from this study contribute to advancing CAM formulations as resilient, eco-friendly materials, offering practical solutions for sustainable infrastructure development.

## Figures and Tables

**Table 1:** Properties of asphalt emulsion, bio-oil, and polymer additives used in CAM formulations.

Property	Asphalt Emulsion	Bio-Oil from Agricultural Waste	Polymer Additives (Poly(styrene-butadiene) Latex and Poly(methyl methacrylate) Acrylic)
Type	Anionic asphalt emulsion	Pyrolysis derived from agricultural waste	Poly(styrene-butadiene) latex and poly(methyl methacrylate) acrylic
Solid Content (%)	45–50%	20–30%	50–60%
Viscosity (Pa·s)	0.5–0.7	0.15–0.2	1.0–1.5
pH	6.5–7.5	5–6 (mildly acidic)	6.0–8.5
Specific Gravity	1.01–1.05	0.90–0.95	1.01–1.10
Tensile Strength Impact	Enhances flexibility, reduces cracking	Improves shrinkage resistance and durability	Increases adhesion and flexibility
Thermal Stability	Stable up to 60 °C	Decomposes at temperatures > 250 °C	Tg: 0–5 °C (for latex)
Chemical Resistance	Moderate resistance to acids and alkalis	High hydrophobicity, reduces water absorption	Excellent resistance to chemicals
Glass Transition (Tg)	N/A	N/A	0–5 °C (for latex)
Environmental Impact	Petrochemical-based, moderate sustainability	Renewable, low-carbon footprint, supports circular economy	Low to moderate sustainability
Primary Function	Enhances flexibility and vibration resistance	Improves workability, reduces shrinkage	Enhances adhesion, flexibility, and durability
Application Temperature	10–50 °C	20–40 °C	0–40 °C

**Table 2:** Mix design of CAM mixtures.

Mixture ID	Cement (%)	Asphalt Emulsion (%)	Bio-Oil (% by Emulsion Weight)	Polymer Additive (%)	Water (%)	Sand (%)	Superplasticizer (%)	Defoaming Agent (%)
M1	75	25	2	0	40	50	2	0.1
M2	75	25	2	1	40	50	2	0.1
M3	75	25	2	1.5	40	50	2	0.1
M4	75	25	2	2	40	50	2	0.1
M5	75	25	4	0	40	50	2	0.1
M6	75	25	4	1	40	50	2	0.1
M7	75	25	4	1.5	40	50	2	0.1
M8	75	25	4	2	40	50	2	0.1
M9	50	50	2	0	40	50	2	0.1
M10	50	50	2	1	40	50	2	0.1
M11	50	50	2	1.5	40	50	2	0.1
M12	50	50	2	2	40	50	2	0.1
M13	50	50	4	0	40	50	2	0.1
M14	50	50	4	1	40	50	2	0.1
M15	50	50	4	1.5	40	50	2	0.1
M16	50	50	4	2	40	50	2	0.1

Note: Percentages are listed independently for each component to indicate individual contributions to the mixture. Bio-oil and polymer additive percentages are calculated based on the weight of asphalt emulsion (AE). Values do not sum to 100% as they represent separate proportions rather than relative fractions of a whole.

## Data Availability

Dataset available on request from the authors.

## References

[1] Li Y., Tian C., Fu Y., Tan Y. (2022). Fracture Parameters and Influencing Factors of Cement Asphalt Mortar. Constr. Build. Mater..

[2] Leiben Z., Wang X., Wang Z., Yang B., Tian Y., He R. (2018). Damping Characteristics of Cement Asphalt Emulsion Mortars. Constr. Build. Mater..

[3] Le T.H.M., Lee S.H., Park D.W. (2020). Evaluation on Full-Scale Testbed Performance of Cement Asphalt Mortar for Ballasted Track Stabilization. Constr. Build. Mater..

[4] Song H., Do J., Soh Y. (2006). Feasibility Study of Asphalt-Modified Mortars Using Asphalt Emulsion. Constr. Build. Mater..

[5] He A., Dong Z., Zhang H., Zhang A.A., Qiu S., Liu Y., Wang K.C.P., Lin Z. (2023). Automated Pixel-Level Detection of Expansion Joints on Asphalt Pavement Using a Deep-Learning-Based Approach. Struct. Control Health Monit..

[6] Yadykova A.Y., Ilyin S.O. (2022). Rheological and Adhesive Properties of Nanocomposite Bitumen Binders Based on Hydrophilic or Hydrophobic Silica and Modified with Bio-Oil. Constr. Build. Mater..

[7] Xue Y., Liu C., Qu J., Lv S., Ju Z., Ding S., An H., Ren K. (2023). Research on Pavement Performance of Recycled Asphalt Mixture Based on Separation Technology of Asphalt and Aggregate in RAP. Constr. Build. Mater..

[8] Foroutan Mirhosseini A., Tahami S.A., Hoff I., Dessouky S., Ho C.H. (2019). Performance Evaluation of Asphalt Mixtures Containing High-RAP Binder Content and Bio-Oil Rejuvenator. Constr. Build. Mater..

[9] Zhang X., Zhang K., Wu C., Liu K., Jiang K. (2020). Preparation of Bio-Oil and Its Application in Asphalt Modification and Rejuvenation: A Review of the Properties, Practical Application and Life Cycle Assessment. Constr. Build. Mater..

[10] Podolsky J.H., Buss A., Williams R.C., Hernández N., Cochran E.W. (2017). Rejuvenation of Vacuum Tower Bottoms through Bio-Derived Materials for Use in Paving Flexible Roadways. J. Clean. Prod..

[11] He L., Tao M., Liu Z., Cao Z., Zhu J., Gao J., Bergh W.V.D., Chailleux E., Huang Y., Vasconcelos K. (2023). Biomass Valorization toward Sustainable Asphalt Pavements: Progress and Prospects. Waste Manag..

[12] Baek C., Lee S. (2018). Laboratory Performance Evaluation of HMA Modified with Various Types and Contents of Polymer. Adv. Civ. Eng..

[13] Bocci M., Grilli A., Cardone F., Ferrotti G. (2014). Full-Depth Reclamation for the Rehabilitation of Local Roads: A Case Study. Int. J. Pavement Eng..

[14] Hamid A., Baaj H., El-Hakim M. (2022). Rutting Behaviour of Geopolymer and Styrene Butadiene Styrene-Modified Asphalt Binder. Polymers.

[15] Ji H., Li B., Li A., Li Z., Han J., Liu Z. (2023). Adhesion Mechanism of Warm-Mixed Recycled SBS Modified Asphalt Binder: Surface Free Energy, Microstructure and Chemical Compositions. Case Stud. Constr. Mater..

[16] Yan K., Lan H., Duan Z., Liu W., You L., Wu S., Miljković M. (2021). Mechanical Performance of Asphalt Rejuvenated with Various Vegetable Oils. Constr. Build. Mater..

[17] Alsolieman H.A., Babalghaith A.M., Memon Z.A., Al-Suhaibani A.S., Milad A. (2021). Evaluation and Comparison of Mechanical Properties of Polymer-Modified Asphalt Mixtures. Polymers.

[18] Ma C., Zhao X., Shi J., Tao J., Zhou H., Dong B., Du Y. (2023). A Rapid-Hardening Cement Emulsified Asphalt (CEA) Mortar Prepared from Magnesium Phosphate Cement. J. Build. Eng..

[19] Wang J., Li X., Sun G., Ma X., Du H. (2024). Experimental Investigation on the Performance of SBS-Modified Asphalt Waterproofing Membrane by Thermo-Oxidative Aging and Freeze–Thaw Cycle. Polymers.

[20] Wang M., Wang Y., Guo J., Xing C., Zou L., Tian S. (2024). Recognition and Characterization of Nanoscale Phases: Modulus Mapping of Asphalt Film in Pavement Mixture Cores. Polymers.

[21] Ouyang J., Hu L., Li H., Han B. (2018). Effect of Cement on the Demulsifying Behavior of Over-Stabilized Asphalt Emulsion during Mixing. Constr. Build. Mater..

[22] Ouyang J., Zhao J., Tan Y. (2018). Modeling Mechanical Properties of Cement Asphalt Emulsion Mortar with Different Asphalt to Cement Ratios and Temperatures. J. Mater. Civ. Eng..

[23] Meng Y., Ouyang J., Ou J. (2023). Investigation on the wetting behavior of asphalt emulsion on aggregate for asphalt emulsion mixture. Constr. Build. Mater..

[24] Tan Y., Ouyang J., Lv J., Li Y. (2013). Effect of Emulsifier on Cement Hydration in Cement Asphalt Mortar. Constr. Build. Mater..

[25] Tan Y., Ouyang J., Li Y. (2014). Factors Influencing Rheological Properties of Fresh Cement Asphalt Emulsion Paste. Constr. Build. Mater..

[26] Le T.H.M., Park D.W., Seo J.W. (2019). Evaluation on the Mechanical Properties of Cement Asphalt Mortar with Quick Hardening Admixture for Railway Maintenance. Constr. Build. Mater..

[27] Le T.H.M., Lee S.H., Park D.W., Seo J.W. (2021). Investigation on the Performance of Railway Ballast Track Stabilized by Cement Asphalt Mortar. Transportation Infrastructure Engineering, Materials, Behavior and Performance, Proceedings of the 6th GeoChina International Conference on Civil & Transportation Infrastructures: From Engineering to Smart & Green Life Cycle Solutions, Nanchang, China, 19–21 July 2021.

[28] Seah C.C., Tan C.H., Arifin N.A., Hafriz R.S.R.M., Salmiaton A., Nomanbhay S., Shamsuddin A.H. (2023). Co-Pyrolysis of Biomass and Plastic: Circularity of Wastes and Comprehensive Review of Synergistic Mechanism. Results Eng..

[29] Siswanto H. (2017). The Effect of Latex on Permanent Deformation of Asphalt Concrete Wearing Course. Procedia Eng..

[30] Lu C.-T., Kuo M.-F., Shen D.-H. (2009). Composition and Reaction Mechanism of Cement–Asphalt Mastic. Constr. Build. Mater..

[31] (2010). Standard Specification for Flow Table for Use in Tests of Hydraulic Cement 1.

[32] (2003). Standard Test Method for Compressive Strength of Cylindrical Concrete Specimens 1.

[33] (2017). Standard Test Method for Length Change of Hardened Cement Mortar and Concrete.

[34] Ye Q., Yang Z., Lv S., Jin J., Zhang S. (2023). Study on Components Selection and Performance of Bio-Oil Based Asphalt Rejuvenator Based on Softening and Asphaltene Deagglomeration Effect. J. Clean. Prod..

